# Supplementary material for: Integration of Cell-Free DNA End Motifs and Fragment Lengths Can Identify Active Genes in Liquid Biopsies
Source: Int J Mol Sci. 2024 Jan 19;25(2):1243. doi: 10.3390/ijms25021243 (PMC10815977; doi:10.3390/ijms25021243)
Supplement: Supplementary file 1 [file ijms-25-01243-s001.zip › ijms-2797735-supplementary.pdf]

Supplementary figures

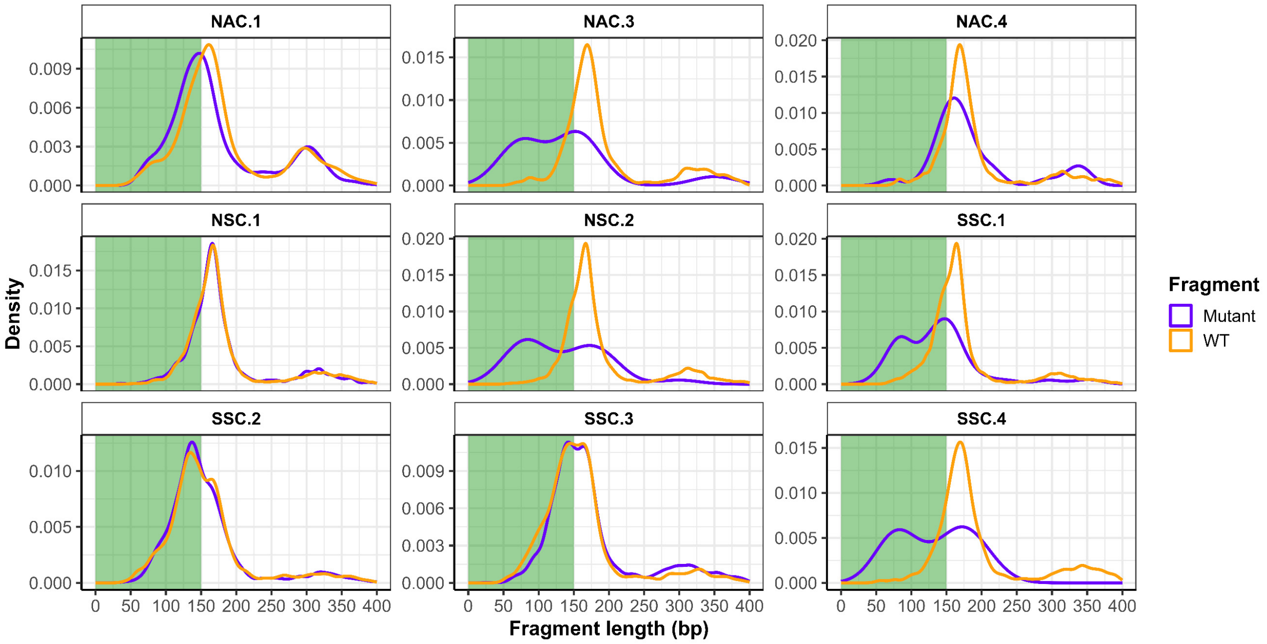

Figure S1. Fragment length distribution of mutant fragments (purple) and WT cfDNA (orange) for individual lung cancer patients. Short cfDNA (0-150 bp) is marked in green.

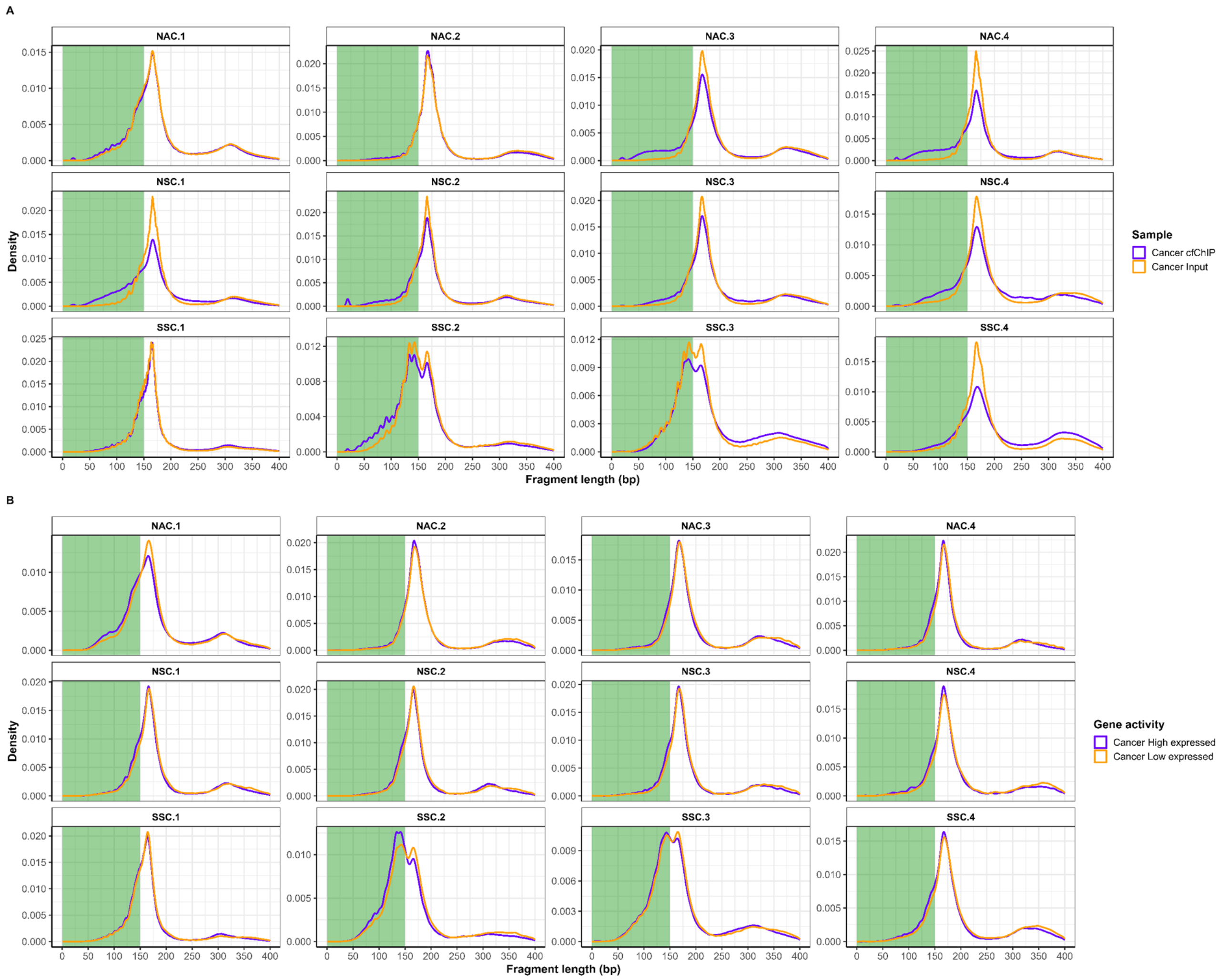

Figure S2. (**A**) cfDNA fragment size distribution in individual lung cancer patient cfChIP samples and input samples. Short cfDNA (0-150 bp) is marked in green. (**B**) cfDNA fragment size distribution in high-expressed (purple) and low-expressed (orange) genes for individual lung cancer patients.

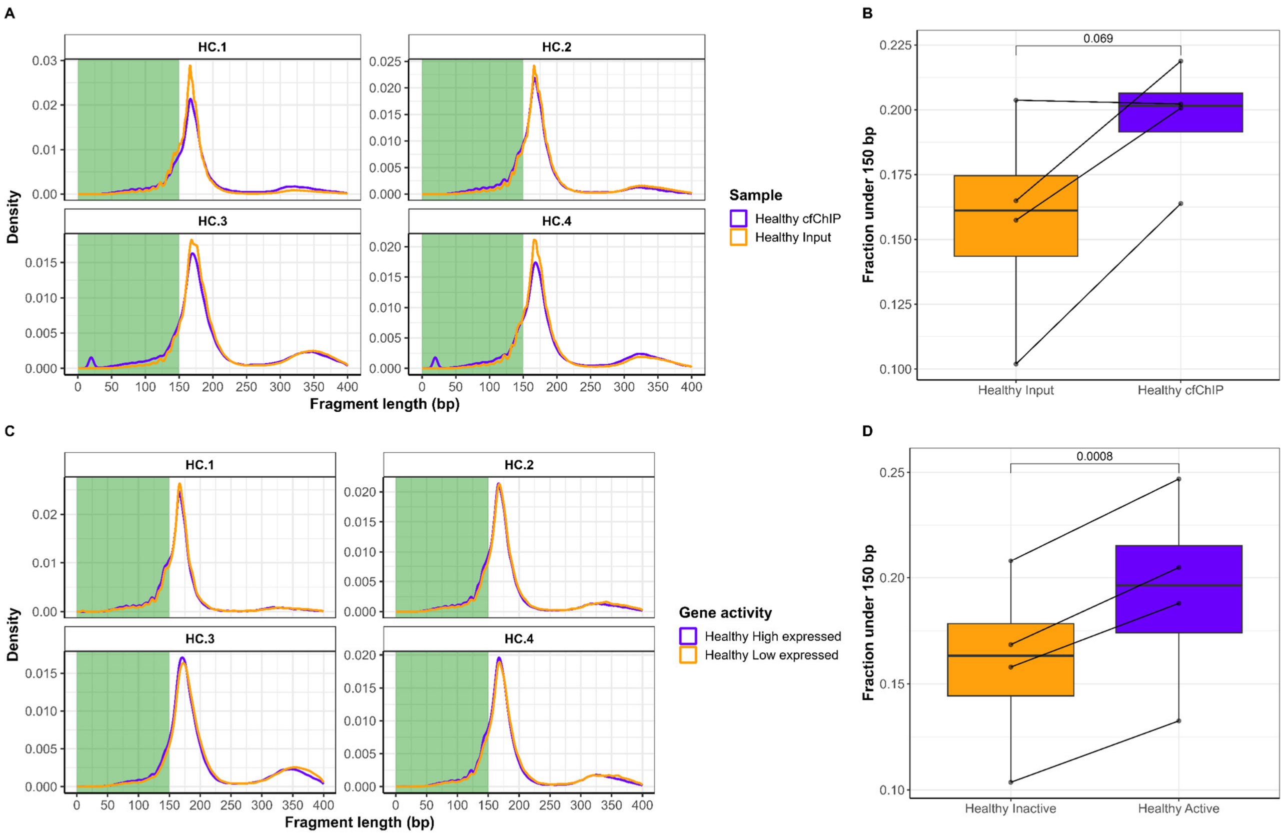

Figure S3. (**A**) cfDNA fragment size distribution in healthy individuals cfChIP samples and input samples. Short cfDNA (0-150 bp) is marked in (**B**) Pairwise comparison of the fraction under 150 bp for input (*n* = 4, orange) and cfChIP (*n* = 4, purple) samples. (**C**) cfDNA fragment size distribution in high-expressed (purple) and low-expressed (orange) genes in healthy individuals. (**D**) Pairwise comparison of the fraction under 150 bp for low-expressed (*n* = 4, orange) and high-expressed (*n* = 4, purple) genes. For (**B**) and (**D**) groups are compared using a paired *t*-test.

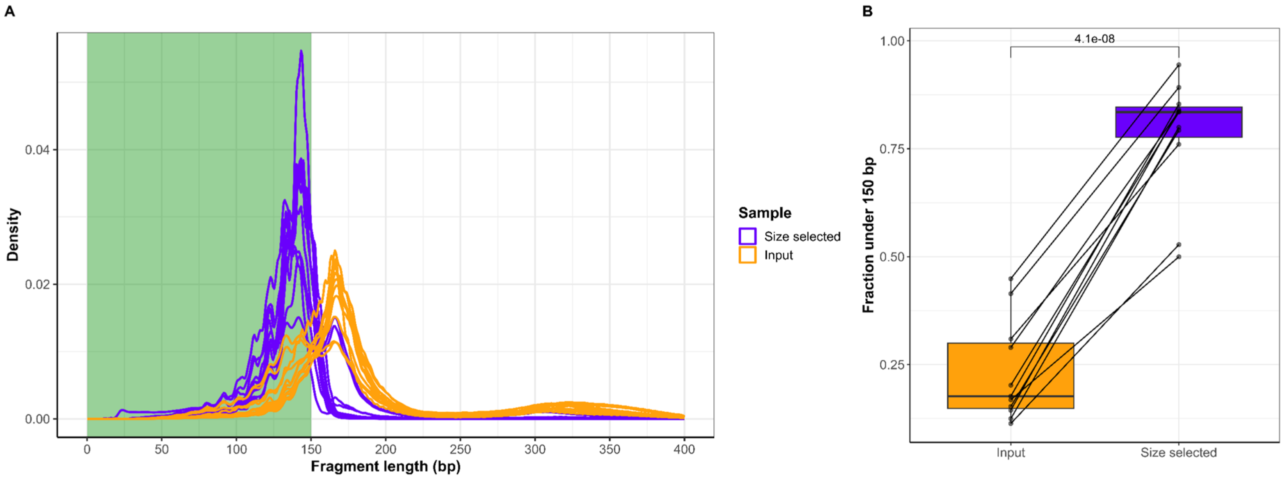

Figure S4. (**A**) cfDNA fragment size distribution in unselected input samples (orange, *n* = 11) and *in vitro* size-selected samples (purple, *n* = 11). Short cfDNA (0-150 bp) is marked in green. (**B**) Pairwise comparison of the fraction under 150 bp for unselected (*n* = 11, orange) and *in vitro* size-selected (*n* = 11, purple) samples. Groups are compared using a paired *t*-test.

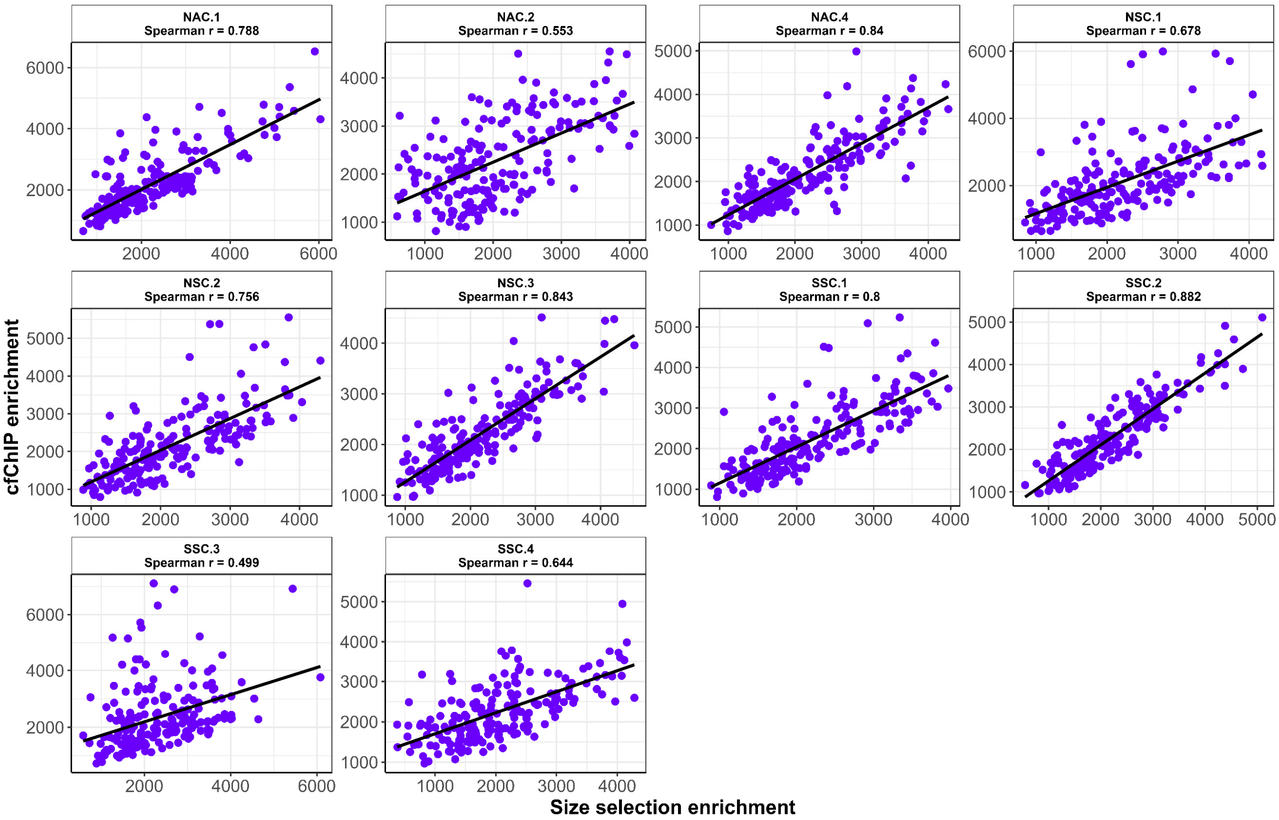

Figure S5. (**A**) Correlation between cfChIP enrichment and *in vitro* size-selection enrichment for 10 lung cancer patients. For each patient, Spearman's correlation between the types of gene enrichment is estimated.

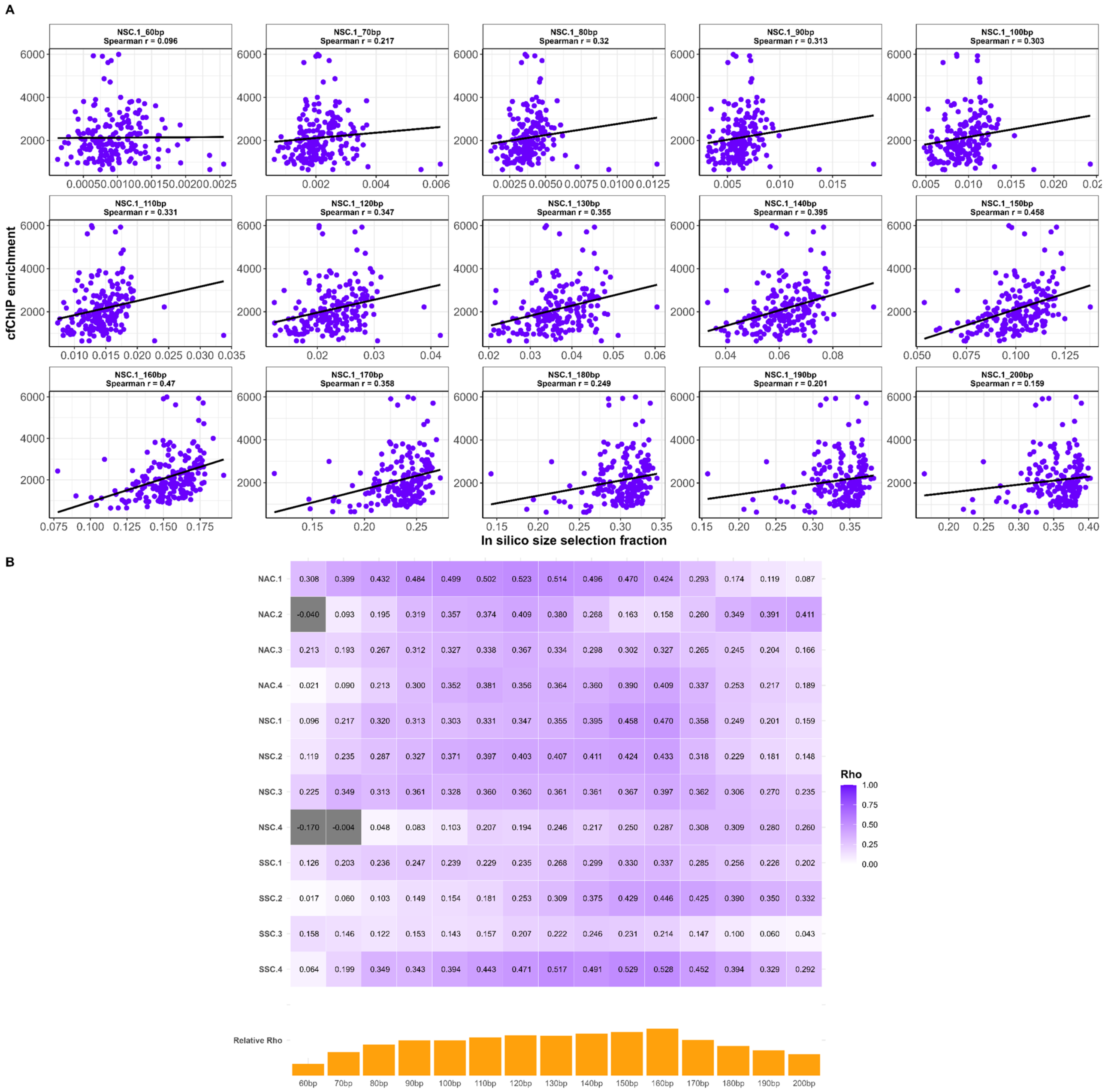

Figure S6. (**A**) Representative example of the correlation between *in silico* size-selection at different cutoffs and cfChIP enrichment. The fraction of input cfDNA fragments with lengths below different cutoffs was compared to the cfChIP gene enrichment from the same patient. The Spearman's r was estimated at every cfDNA fragment length cutoff. (**B**) Spearman r for each cfDNA fragment cutoff compared to cfChIP enrichment for 12 lung cancer patients. The median Spearman's r for each fragment length cutoff relative to the global Spearman's r of all comparisons is represented with orange bars.

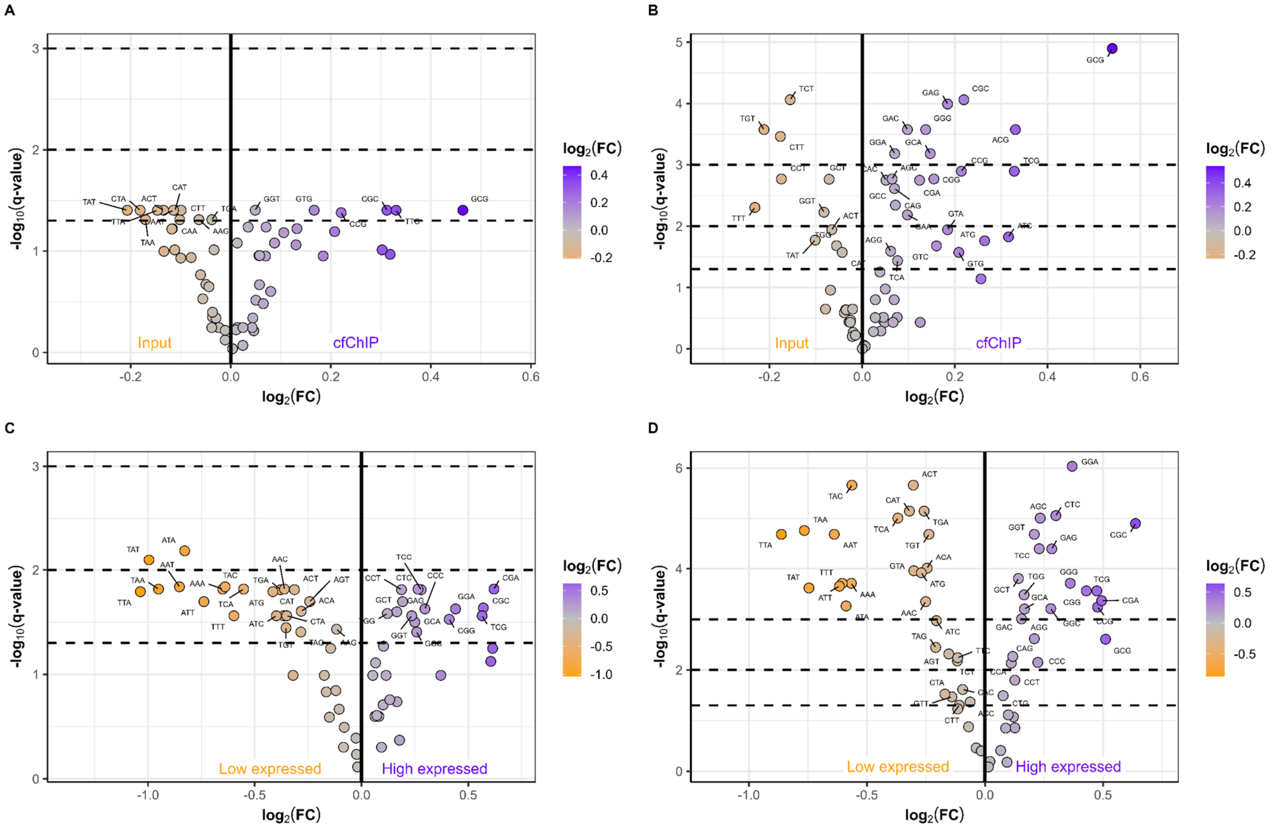

Figure S7. (**A**) and (**B**) Differences in fragment end motifs in paired input and cfChIP. (**A**) for healthy individuals, *n* = 4, (**B**) for all samples, *n* = 16. (**C**) and (**D**) Differences in fragment end motifs for low/high expression genes. (**C**) for healthy individuals, *n* = 4, (**D**) for all samples, *n* = 16.

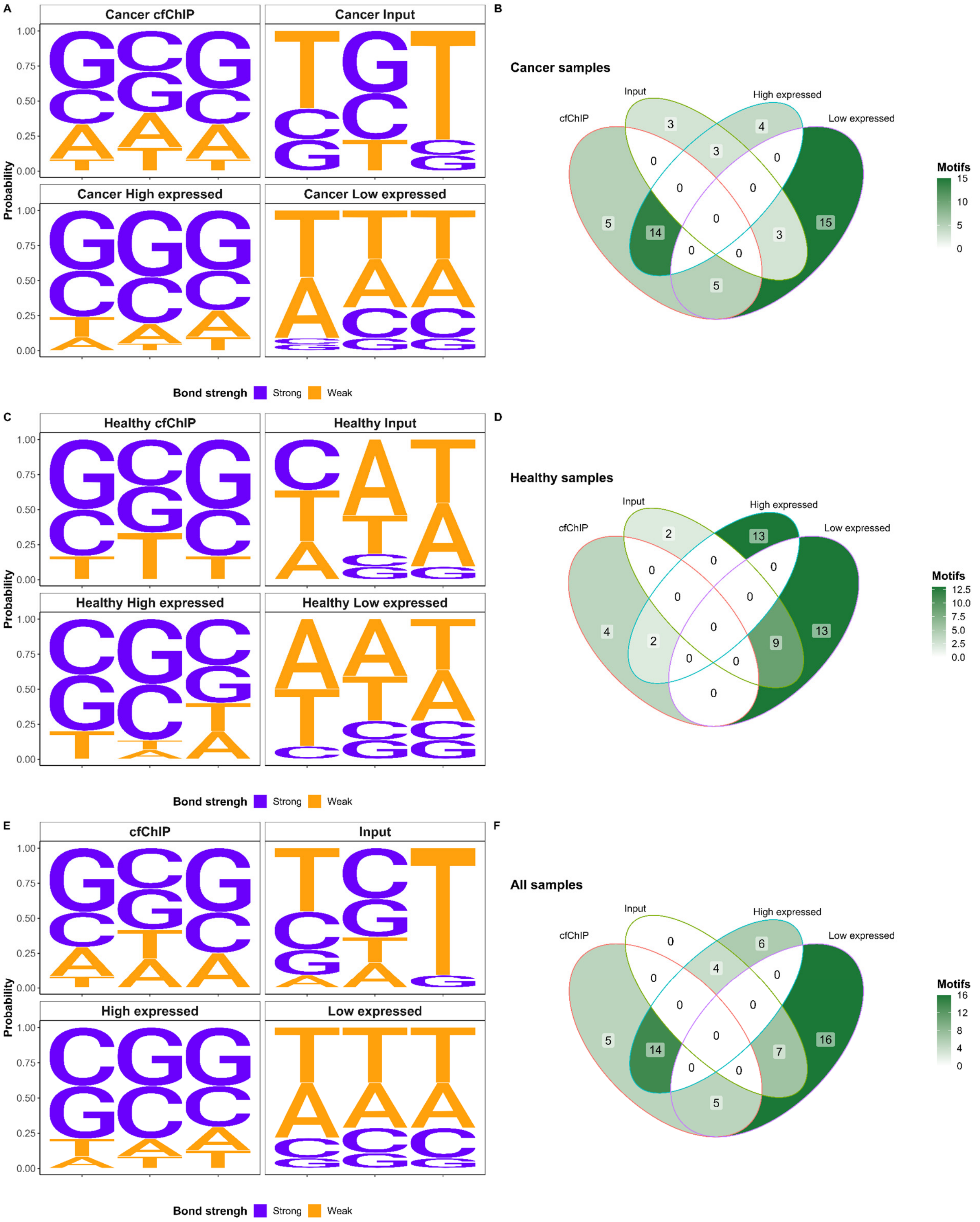

Figure S8. (**A**), (**C**), and (**E**) Consensus sequence between significant (q < 0.05) fragment end motifs upregulated in cfChIP samples, input samples, high-expressed genes, or low-expressed genes. (**A**) Cancer patients, *n* = 12, (**C**) Healthy individuals, *n* = 4, (**E**) All individuals, *n* = 16. (**B**), (**D**), and (**F**) Venn diagrams representing the overlap in significant fragment end motifs in the analyses of cfChIP compared to input samples and high-expressed compared to low-expressed genes. (**B**) Cancer patients, *n* = 12, (**D**) Healthy individuals, *n* = 4, (**F**) All individuals, *n* = 16.

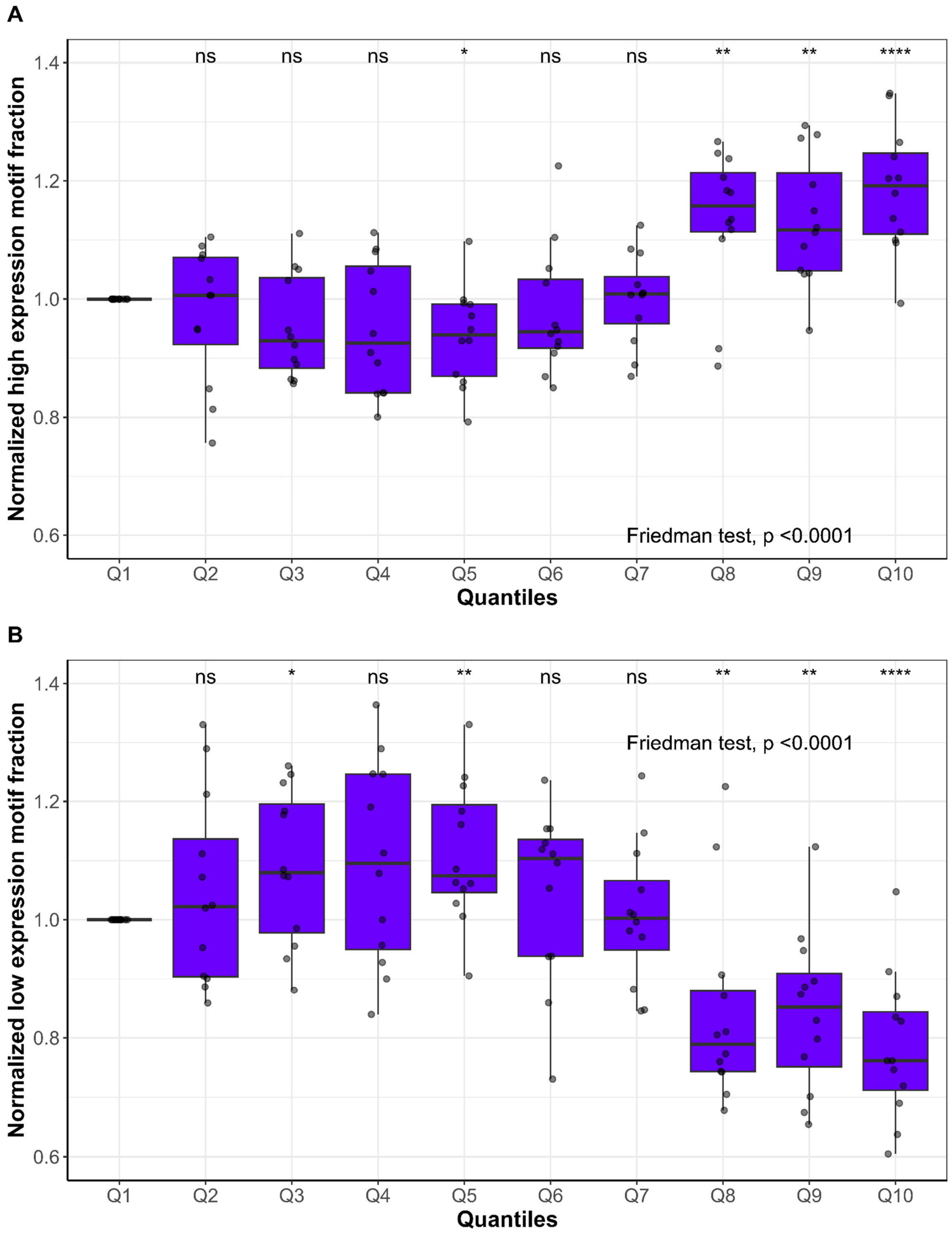

Figure S9. For each lung cancer patient *n* = 12 the genes in cfChIP quantiles are determined. For each quantile the fraction of fragments with fragment end motifs upregulated in high-expressed genes (**A**) or low-expressed genes (**B**) samples is calculated. Each quantile is normalized to the fraction in Q1.

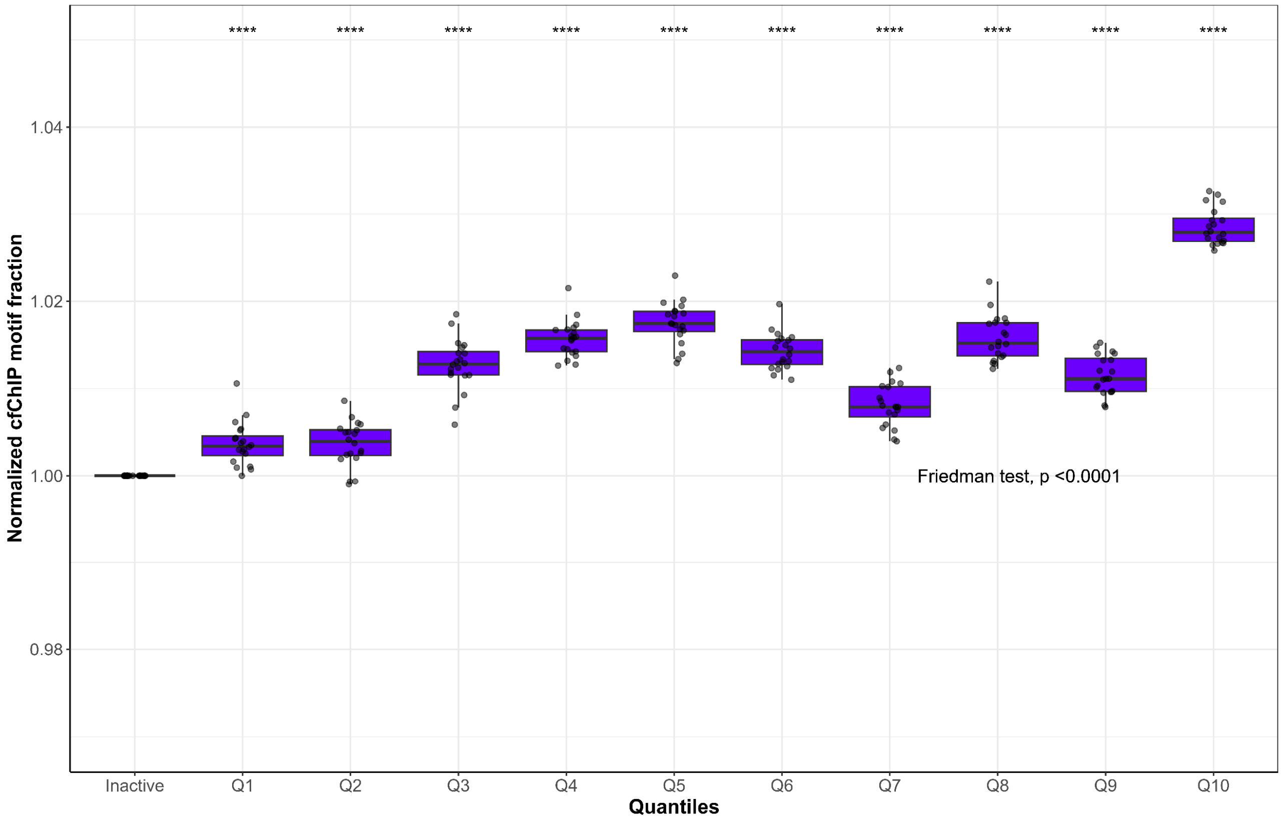

Figure S10. For each sample in the validation dataset (*n* = 20) genes are grouped in quantiles according to gene expression in PBMCs. For each quantile the fraction of fragments with fragment end motifs upregulated cfChIP-samples. Each quantile is normalized to the fraction in inactive genes.

Supplementary tables

Table S1. Plasma volume used for input cell-free DNA, cell-free chromatin immunoprecipitation, and in vitro size-selection. The resulting number of position deduplicated reads and mean unique read depths are also given

Sample	Plasma used as input (mL)			No. Position deduplicated reads			Mean unique read depth		
	Input	cfChIP	Size-selected	Input	cfChIP	Size-selected	Input	cfChIP	Size-selected
NAC.1	0.5	3.3	1.7	10,502,644	1,497,783	1,688,002	1,799	218	351
NAC.2	0.5	3.3	1.9	7,229,193	1,448,09	1,251,643	929	217	369
NAC.3	0.5	3.2	1.9	4,412,085	433,742	160,370	581	60	26
NAC.4	0.5	3.0	1.8	5,028,413	1,327,089	236,960	655	175	46
NSC.1	0.5	3.1	1.8	9,547,530	928,203	1,622,035	1,536	80	230
NSC.2	0.5	3.2	1.7	7,115,997	402,093	680,070	863	58	101
NSC.3	0.5	3.4	1.9	3,004,381	705,889	614,856	261	126	108
NSC.4	0.5	3.1	NA	1,946,282	330,812	NA	189	66	NA
SSC.1	0.5	3.0	1.8	18,005,689	6,846,579	8,100,620	3,731	1,185	1,425
SSC.2	0.5	3.3	1.5	7,538,219	1,955,738	2,330,012	1,186	288	417
SSC.3	0.5	3.0	1.8	10,203,132	3,262,739	2,869,449	1,690	597	616
SSC.4	0.5	3.2	1.9	8,528,750	1,222,672	1,370,764	807	181	392
HC.1	1.0	3.0	NA	3,127,460	200,702	NA	457	19	NA
HC.2	1.0	3.0	NA	4,435,973	140,821	NA	578	24	NA
HC.3	1.0	3.0	NA	4,287,776	161,028	NA	522	23	NA
HC.4	1.0	3.0	NA	3,425,299	132,783	NA	579	22	NA
HC.5	1.0	NA	NA	2,183,415	NA	NA	302	NA	NA
HC.6	1.0	NA	NA	4,265,228	NA	NA	365	NA	NA
HC.7	1.0	NA	NA	5,389,182	NA	NA	673	NA	NA
